# 

**Published:** 1834-06-01

**Authors:** 


### GENERAL INDEX

TO THE

NEW SERIES

OF THE

### MEDICO-CHIRURGICAL REVIEW,

**VOLUME I. to VOLUME XX.**

INCLUSIVE,

[From JUNE 1st, 1824, to JUNE 1st, 1834.]

---

### With an Appendix,

COMPRISING

### AN INDEX

TO

THE SERIES OF FOUR ANNUAL VOLUMES,

FROM

**JUNE 1820 to APRIL 1824.**

Surgeon General's Office
LIBRARY.
57460.
Washington, D.C.

LONDON:

PUBLISHED BY S. HIGHLEY, 32, FLEET STREET.

1834.

W1
ME75T

PRINTED BY G. HAYDEN,
Little College Street, Westminster.

